# Changes in soil bacterial and fungal communities in response to *Bacillus megaterium* NCT-2 inoculation in secondary salinized soil

**DOI:** 10.7717/peerj.12309

**Published:** 2021-10-12

**Authors:** Bin Wang, Shaohua Chu, Xiaorui Liu, Dan Zhang, Xiaotong Chai, Xijia Yang, Yuee Zhi, Yaowei Chi, Pei Zhou

**Affiliations:** 1 School of Agriculture and Biology, Shanghai Jiao Tong University, Shanghai, China; 2 Key Laboratory of Urban Agriculture, Ministry of Agriculture and Rural Affairs, Shanghai, China; 3 Bor S. Luh Food Safety Research Center, Shanghai Jiao Tong University, Shanghai, China

**Keywords:** *Bacillus megaterium* NCT-2, Nitrate, Microbial community, FUNGuild, Ectomycorrhizal fungi

## Abstract

**Background:**

Secondary salinized soil in greenhouses often contains excess nitrate. Inoculation of *Bacillus megaterium* NCT-2 with nitrate assimilation ability represents an attractive approach for soil remediation. However, the effects of NCT-2 on the structure and function of soil microbial communities have not been explored.

**Methods:**

Greenhouse experiments were carried out to investigate changes in soil properties, *Brassica chinensis* L. growth, bacterial, and fungal community structure and function in response to NCT-2 inoculation.

**Results:**

The NCT-2 inoculant significantly reduced the nitrate content in *B. chinensis* and inhibited the rebound of soil nitrate in the later stage. The shifts of bacterial community structure and function by NCT-2 was negligible, and a greater disturbance of soil fungal community structure and function was observed, for example the strong inhibitory effect on ectomycorrhizal fungi. These results indicated that the NCT-2 inoculant likely achieved the remediation effect in secondary salinized soil by shifting fungal community. The present findings add to the current understanding of microbial interactions in response to bacterial inoculation and can be of great significance for the application of NCT-2 inoculants in secondary salinized soil remediation.

## Introduction

Soil salinization is undisputedly regarded as a major threat to agricultural soil, because it has direct adverse effects on soil quality (Ammari et al., 2013; Daliakopoulos et al., 2016). Soil salinity is resulted from several ways (Otlewska et al., 2020)—primarily through changes in natural conditions or secondarily *via* excessive use of fertilizers (Datta & Jong, 2002). The overuse of both chemical and organic nitrogen fertilizers can result in an increase of soil nitrate content indicated by an increase in electrical conductivity (EC) or by signs of crop disease related to severe soil salinization (Gkiougkis et al., 2015; Otlewska et al., 2020). In consideration of the significance of agricultural production, it is important to develop effective approaches for amending salt-affected soil (Otlewska et al., 2020). The salt-resistance genes of halophytes can be expressed in conventional crops, however, this strategy has proved to be inefficient and was implemented mainly under laboratory conditions (Otlewska et al., 2020). In addition, the pre-treatment of salinity resistant plants with chemicals like 5-aminolevulinic acid (El-Esawi et al., 2018), or with physical effectors like UV-B irradiation (Dhanya Thomas, Dinakar & Puthur, 2020) for the production of salinity resistant plants is environmentally unfriendly and not recommended for sustainable agriculture (Otlewska et al., 2020), due to the possible pollution caused by additive chemicals or ultraviolet ray. By using soil microbial species with colonization and plant growth promoting ability in salinized environment represents a promising approach for enhancing the agricultural production of salinized land (Otlewska et al., 2020). Further, microbial inoculants offer a cheaper alternative to fertilizers for small-holder farmers (Ambrosini, de Souza & Passaglia, 2016).

The application of bacterial inoculants is an attractive alternative that would substantially reduce the use of chemical fertilizers and pesticides, with an increasing number of inoculants being commercialized for various crops (Berg, 2009). To harbor the benefits of this approach, a high concentration of inoculant must be introduced into the environment (Lupwayi et al., 2000). However, the potential environmental impacts related to inoculation have always been neglected (Trabelsi & Mhamdi, 2013). Since inoculation introduces high densities of viable and functionally active microbes for rapid colonization of the host rhizosphere, a transient perturbation of soil microbial community equilibrium is to be expected (Trabelsi & Mhamdi, 2013). A potential loss of important native species would be highly undesirable, affecting subsequent crops (Trabelsi & Mhamdi, 2013).

There is great variability amongst results of bacterial inoculation in different plant and soil conditions (Ambrosini, de Souza & Passaglia, 2016). Some studies reported no effects or a transient effect, while others demonstrated long-term changes (Trabelsi & Mhamdi, 2013). Some beneficial bacteria can provide nutrients and phytohormones for plants to help them resist salt stress (Otlewska et al., 2020). These growth-promoting and protective effects are not necessarily a direct result of strain inoculation and may be related to the induction or repression of resident microbial populations (Trabelsi & Mhamdi, 2013). Understanding the underlying mechanisms of inoculants will allow us to focus on beneficial species to be used as such (Otlewska et al., 2020).

Moreover, understanding the function of specific microbes could allow for the development of more precise management practices leading to improved sustainability and productivity in agricultural ecosystems (Nie et al., 2018). As a result of the rapid development of high-throughput sequencing, the entire microbial community can now be extensively and accurately characterized (Chen, Wang & Wen, 2018). On the basis of data from high-throughput sequencing, PICRUSt and FUNGuild have been explored as viable tools for functional prediction of bacterial and fungal communities (Langille et al., 2013; Toju et al., 2016).

A novel bacterium, *Bacillus megaterium* NCT-2, which was isolated and identified in secondary salinization soil obtained from greenhouses in China (Shi et al., 2014), exhibited high nitrate reduction capacity (Chu et al., 2017) and plant growth-promoting activity (Chu et al., 2018). To the best of our knowledge, little information is available regarding the time course of changes in the structure and function of bacterial and fungal communities following bacterial inoculation in secondary salinization soils. Knowledge on the alterations in fungal community composition and function in response to bacterial inoculation in secondary salinization soil is particularly limited. A detailed picture of how microbial communities specifically respond to *B. megaterium* NCT-2 inoculation is therefore lacking, which limits our understanding of its potential for secondary salinization soil amendment. Therefore, we hypothesize that NCT-2 inoculation can reduce soil nitrate content and promote plant growth by shifting the bacterial and fungal community in secondary salinized soil. In the present study, we determined the impacts of NCT-2 inoculation on soil properties and crop productivity, and used 16S rRNA gene amplicon sequencing, ITS gene amplicon sequencing, and functional analysis in order to analyze the responses of microbial community structure and function to NCT-2 inoculation in secondary salinization soil.

## Materials and Methods

### Preparation of microbial agent

The nitrate-assimilating bacterium *Bacillus megaterium* NCT-2 (CGMCC No. 4698), previously isolated from secondary salinization soil; was incubated at 35 °C for 48 h in a 100 L fermenter (BLBIO-100SC; Shanghai Bailun Bio-Technology Co., Ltd., Shanghai, China) containing 70 L of culture medium (yeast extract 3.0 g L^−1^, sucrose 15.0 g L^−1^, KH_2_PO_4_ 0.5 g L^−1^, MnSO_4_ 0.05 g L^−1^, pH 7.2). The aeration rate and agitation speed were controlled at 2.0 vvm and 200 rpm, respectively. The microbial agent was then produced by mixing bacterial fermentation culture liquid and straw powder with an optical live bacteria number of 2  × 10^8^ CFU g^−1^.

### Study site and greenhouse experimental design

The field study was conducted at a greenhouse in Jiading district, Shanghai, China (121°14′E, 31°37′N), which had secondary salinization soil. The field was divided into nine plots, each 4 m × 20 m in size. Three treatments were separately applied to plots before seeding *Brassica chinensis* L*.*: (1) microbial agent (45 g m^−2^) was homogenized into soil to a depth of up to 15 cm (NCT treatment, *n* = 3); (2) straw powder (45 g m^−2^) with liquid media was used (straw treatment, *n* = 3); (3) neither microbial agent or straw power was used (control treatment, *n* = 3). Each treatment consisted of three plots designed as three replications. *B. chinensis* seedlings were grown in nursery pots, after 10 days, the seedlings were transplanted into the secondary salinized soil, under the same field management and environmental conditions. After 72 days, *B. chinensis* plants were harvested.

### Soil sampling

Bulk soil cores of 0–20 cm depth were collected using the “S” sampling method prior to soil treatments as well as on days 7, 14, 28, and 72 of sowing. In consideration of the perturbation of soil microbial community following NCT treatment, the samples were taken frequently in the first month. And then the soil samples were collected after harvest (72d) to examine if the effect of NCT-2 inoculant can last until harvest. In detail, nine soil samples were collected from the field before soil treatments, three soil samples of which were sieved (1.0 mm) and homogenized thoroughly into one sample as the samples before treatment (“Before”, *n* = 3). After sowing, three soil samples were randomly collected from plots with each treatment on every sampling day. For NCT treatment and straw treatment, each sample was divided into two parts. One part of the soil sample was used to determine soil physiochemical properties, including nitrate content and enzyme activity. The other was frozen at −80 °C for DNA extraction and subsequent microbial analysis. For control treatment, the soil sample was only used to determine soil physiochemical properties.

### Soil and plant property measurements, and statistical analysis

The subsequent measurements were applied according to *Soil Argrochemistry Analysis Protocoes* (Ru-kun, 1999). Soil electrical conductivity (EC) and pH were determined using a conductivity meter and a pH meter (Sincere Dedication of Science and Technology Innovation, Shanghai, China) at a 1:2.5 ratio (weight/volume, w/v) of soil to distilled water. Organic matter was determined using K_2_Cr_2_O_7_ with an external heating method. To assess the levels of cations and anions, 50 mL deionized water was mixed with 10 g soil samples, followed by incubation at 25 °C and 180 rpm over 15 min in a shaker incubator. The mixture was then passed through a 1.0 mm sieve. The supernatant was stored at 4 °C until detected. Concentrations of Na^+^, K^+^, Ca^2+^, and Mg^2+^ were analyzed using inductively coupled plasma-atomic emission spectrometry (ICP-AES). Cl^−^ content was assayed *via* the precipitation titration method, SO_4_^2−^ content was determined through the EDTA indirect coordination titration method, and HCO_3_^−^ content was analyzed with the double indicator neutralization titration method.

To measure the NO_3_^−^ concentration in soil, 4 g dry weight soil samples were suspended in 20 mL of 2M KCl solution. After shaking at room temperature for 1 h and then being kept at rest for 30 min, the supernatants were filtered and analyzed using a continuous flow analytical system AA3 (BRAN-LUBBE, Hamburg, German). The nitrate content of plants was analyzed according to a standard method as per the national food safety standard of China (GB/T5009.33).

Soil urease activity was determined *via* colorimetry (Weaver & Soil Science Society of America, 1994). Briefly, five grams of soil sample were placed in a 100 mL triangle flask, and one mL toluene was added. Subsequently, the flask was kept at room temperature for about 15 min. Five milliliters of 10% urea solution (w/v) and 10 mL citrate buffer (citric acid 184 g L^−1^, KOH 147.5 g L^−1^, pH = 6.7) were added and incubated at 28 °C for 24 h. One milliliter of the filtered solution and nine mL of distilled water were put into a 50 mL volumetric flask. Four milliliters of sodium phenate solution (1.35 mol L^−1^) and three mL of sodium hypochlorite (0.9%) were added successively, completely mixed, and left for 20 min, diluted with distilled water to 50 mL. Absorbance at 578 nm was then analyzed within 1 h using a spectrophotometer. Urease activity was expressed as µg NH_3_-N g^−1^ h^−1^.

Soil phosphatase activity was determined by quantifying the mg of C_6_H_5_NO_3_ g^−1^ h^−1^ (F. Eivazi, 1977). One gram of soil sample was mixed with 0.2 mL of toluene, four mL of phosphate buffer (pH 6.5), and one mL of p-nitrophenyl phosphate disodium (0.05 M), followed by incubation at 37 °C for 1 h. Thereafter, one mL of 0.5 M CaCl_2_ and four mL of 0.5 M NaOH were added successively. The reaction mixture was centrifuged at 2500 rpm for 5 min, and the supernatant was subjected to spectrophotometry analysis at 410 nm after being further centrifuged at 4000 rpm for 5 min.

The fresh weight and height of six *B. chinensis* plants in each treatment were measured by weighing and estimating all parts of the plant except the roots at the time of harvest. The total production of *B. chinensis* was measured by weighing the fresh weight of all plants in each plot. The vitamin C (Vc) and nitrate content of six plants in each treatment was analyzed according to national standard analytical methods (GB 6195-86 and GB 5009.33-2010, respectively). The chlorophyll content of six plants in each treatment was measured using a chlorophyll meter CCM-300 (OPTI-SCIENCES, Hudson, NH, USA).

### DNA extraction, amplification, and sequencing

Total DNA from 27 soil samples (three soil samples per condition on each sampling day in NCT treatment and straw treatment) were extracted using the UltraClean Soil DNA Isolation Kit (MoBio Laboratories Inc., Carlsbad, CA, USA) as the manufacturer’s instructions. The integrity of extracted DNA was confirmed *via* agarose electrophoresis analysis. DNA quality assessment and quantification were conducted using a Nano-Drop 2000 spectrophotometer (Thermo Scientific, Wilmington, DE, USA). The V4V5 region of bacterial 16S rDNA was amplified using primers 515F (5′- GTGCCAGCMGCCGCGG-3′) and 907R (5′- CCGTCAATTCMTTTRAGTTT-3′). The fungal ITS rRNA gene was amplified with primers ITS1F (5′-CTTGGTCATTTAGAGGAAGTAA-3′) and ITS2 (5′-GCTGCGTTCTTCATCGATGC-3′). PCR reactions were performed in 20- µL reaction mixtures containing 10 ng of DNA template, 0.2 µM of both primers, 0.4 µL of FastPfu polymerase, 4 µL 5 × FastPfu buffer, and 2 µL of 2.5 mM dNTPs. Thermal cycling conditions were as follows: an initial denaturation step at 94 °C for 3 min, followed by 27 cycles of denaturation at 94 °C for 30 s, annealing at 55 °C for 30 s, and extension at 72 °C for 45 s, followed by a final extension step at 72 °C for 10 min. After purification using the AxyPrep DNA gel extraction kit (Axygen Biosciences, Union City, CA, USA) and re-quantification on a QuantiFluorTM-ST (Promega, Madison, WI, USA) system, the purified amplicons were pooled and sequenced (2 ×300 bp paired-end reads) on the MiSeq platform (Illumina, San Diego, CA, USA) at Shanghai Majorbio Bio-pharm Technology Co., Ltd. (Shanghai, China).

### Processing of sequence data and diversity analysis

Sequencing data were uploaded and analyzed *via* the free online Majorbio Cloud Platform (http://www.majorbio.com, accessed on 23 March 2021). The raw reads were demultiplexed into samples based on the barcode with zero mismatches, and quality filtered using QIIME (version 1.9.1) (Caporaso et al., 2010). In details, the reads were truncated at the region with less than 20 quality score over a 50 base pairs (bp) sliding window from the ending. The reads with the length less than 50-bp were discarded. Reads with more than 10-bp overlap were merged. Quality-filtered reads were clustered into operational taxonomic units (OTUs) with similarity greater than 97%. To eliminate the differences in the sequencing depth, all samples were normalized to the smallest sequence number. The most abundant sequence from each OTU was assigned taxonomy using the RDP classifier (http://rdp.cme.msu.edu/, accessed on 23 March 2021) (Wang et al., 2007) against the SILVA (Quast et al., 2013) and UNITE reference databases (Nilsson et al., 2019) for 16S V4V5 and ITS data, respectively. The confidence threshold was set at 70% with default conditions through the pipeline of Majorbio Cloud Platform. The sequences of all samples were sized to the lowest number of reads in any individual sample in order to eliminate differences in sequencing depth. The estimators of alpha-diversity were calculated using Mothur (version 1.30.2). Differences in the entire community composition were determined using the Bray-Curtis distance based on OTU abundance and visualized *via* the non-metric multidimensional scaling (NMDS). The ‘Vegan’ package (Dixon, 2003) in R was used for redundancy analysis (RDA) in order to explore the correlations between microbial communities and environmental factors. All differences were considered significant at *p* < 0.05. The Kyoto Encyclopedia of Genes and Genomes (KEGG) pathways provided by the Phylogenetic Investigation of Communities by Reconstruction of Unobserved States (PICRUSt) were used to predict bacterial function (Langille et al., 2013). The functional profiles of fungi were predicted based on FUNGuild in order to analyze the response of fungal trophic strategies (Nguyen et al., 2016).

### Statistical analysis

Nonparametric test (Mann–Whitney test) was used to determine the difference between two treatments. Data represent means of the biological replicates ± SEM. Results were considered statistically significant at *p* < 0.05.

## Results

### Effect of NCT-2 inoculation on soluble salt content and soil properties

**Figure 1:**
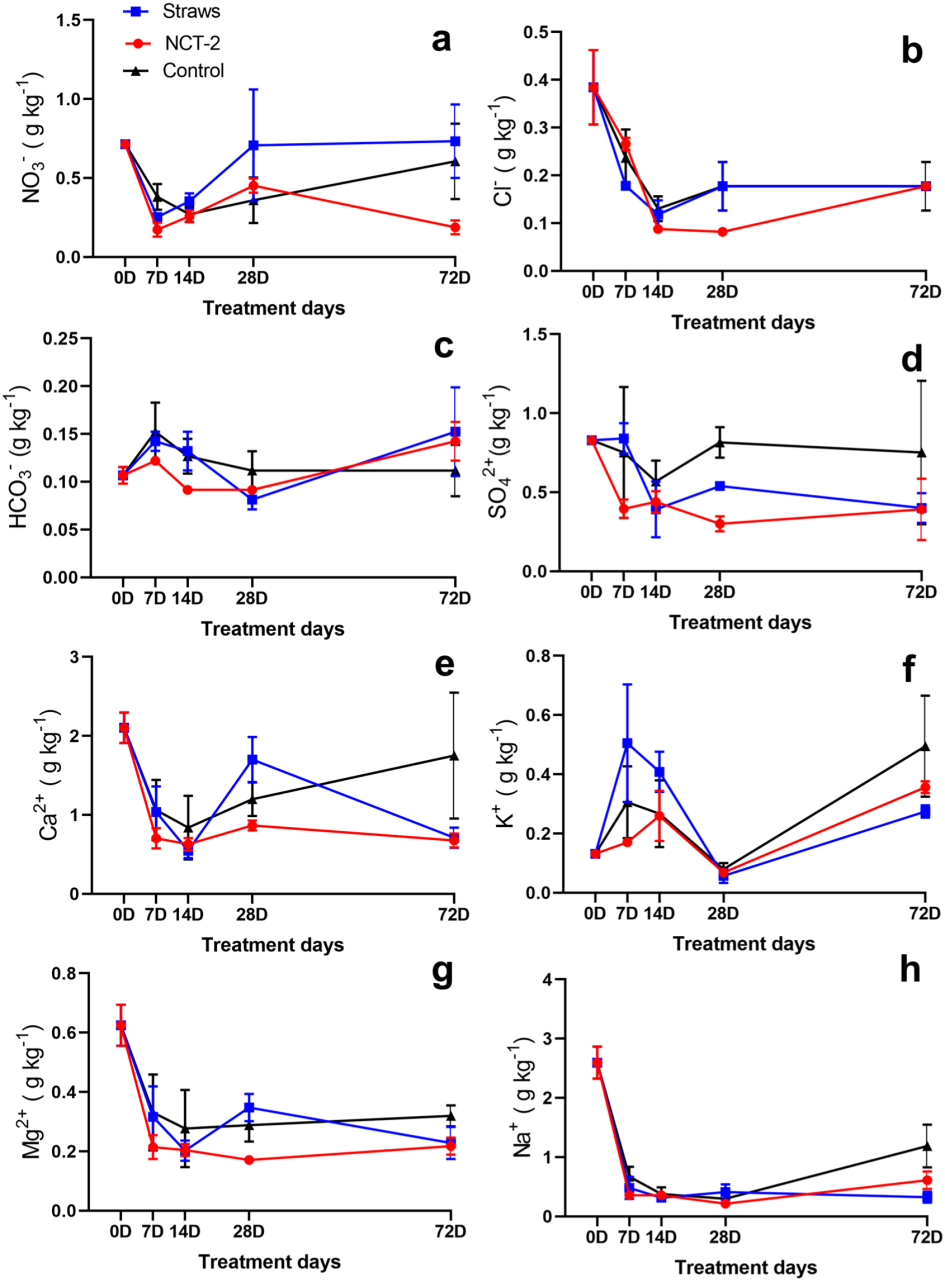
Changes of salt ion content in NCT-2 and straw treatment soil. (A) NO_3_^−^, (B) Cl^−^, (C) HCO_3_^−^, (D) SO_4_^2−^, (E) Ca^2+^, (F) K^+^, (G) Mg^2+^, (H) Na^+^.

As shown in Fig. 1A, a fluctuation in soil nitrate content was observed following both NCT and straw treatment. At the early stage, nitrate content in both groups obviously decreased, which may be mainly attributed to plant absorption. Thereafter, the nitrate content rose slowly. A greater reduction of nitrate content was observed in NCT-treated soil at the end of the experiment, which was just a quarter of nitrate content in straw treatment and one third of nitrate content in soil control treatment. However, the difference in nitrate content between treatments was not significant in each period, which may be related to the large variability among analyzed replicates. The levels of other salt ions, including Cl^−^, HCO_3_^−^, SO_4_^2−^, Ca^2+^, K^+^, Mg^2+^, and Na^+^, also fluctuated in all treatment groups (Figs. 1B–1H), with no significant differences and similar contents observed for any of these on the 72nd day. The content of NO_3_^−^, Cl^−^ and K^+^ in soil all increased again in the late stage of the experiment, nevertheless, only the rebound of nitrate content was suppressed in NCT treatment.

**Figure 2:**
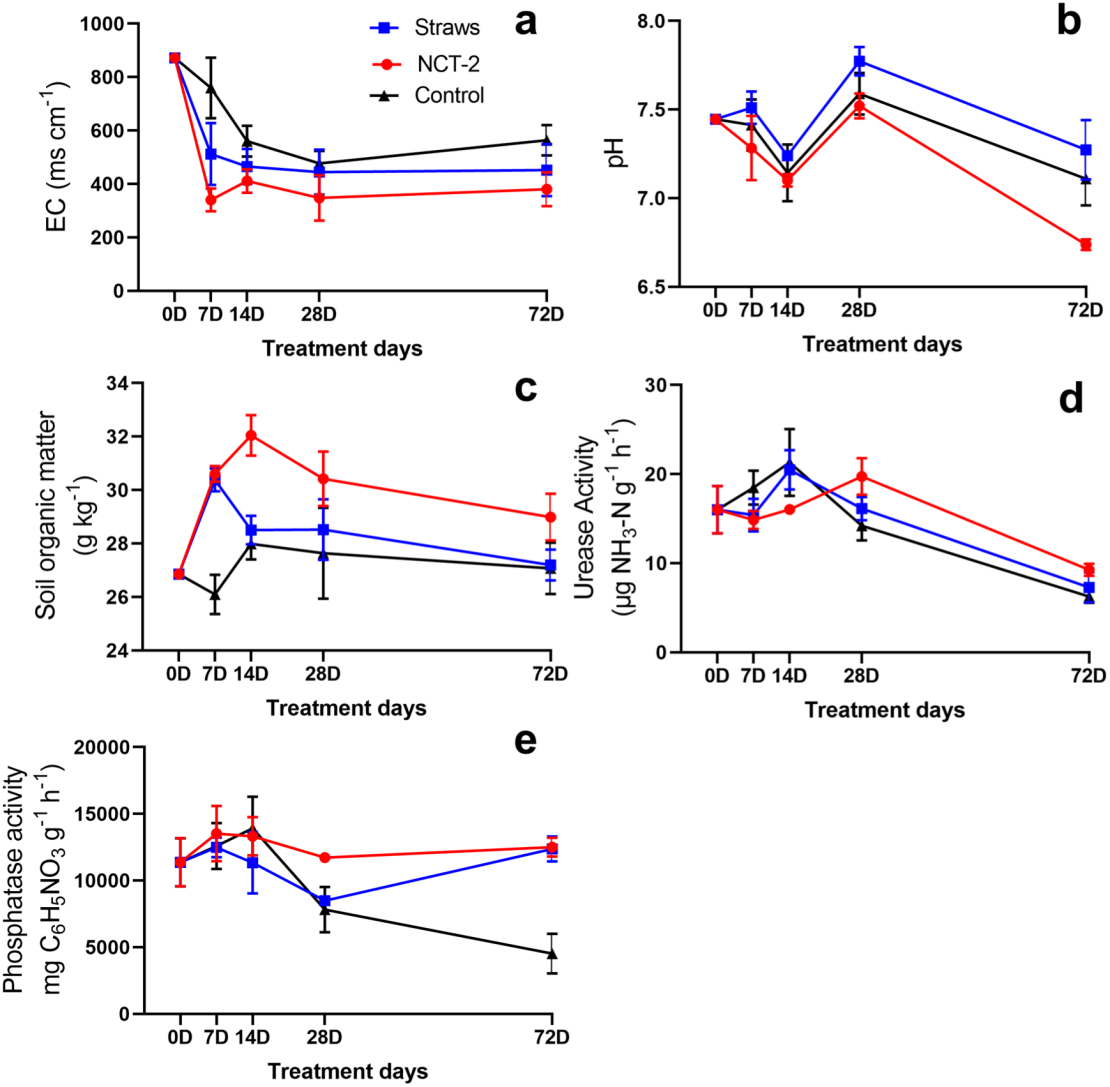
Changes of soil properties in NCT and straws treatment groups. (A) EC, (B) pH, (C) soil organic matter, (D) urease activity, (E) phosphatase activity.

Moreover, compared with straw treatment, NCT-2 inoculation reduced the EC and pH values of soil throughout the experiment (Figs. 2A, 2B). The lower EC value of soil in the NCT treatment group may be caused by the lower nitrate content. Although the pH value of soil was decreased following NCT-2 inoculation, NCT-2 did not cause soil acidification. Further, soil organic matter content in the NCT group remained higher than in the straw powder-treated soil (Fig. 2C), indicating that NCT-2 inoculation can increase soil organic matter. With regard to soil enzymes, the NCT-2 inoculant upregulated urease activity from the 28th day as well as phosphatase activity throughout the experiment (Figs. 2D, 2E). Our results implied that the NCT-2 inoculant could enhance nutrient transformation in soil.

### Effect of NCT-2 inoculation on the nitrate accumulation and growth promotion of *B. chinensis*

**Figure 3:**
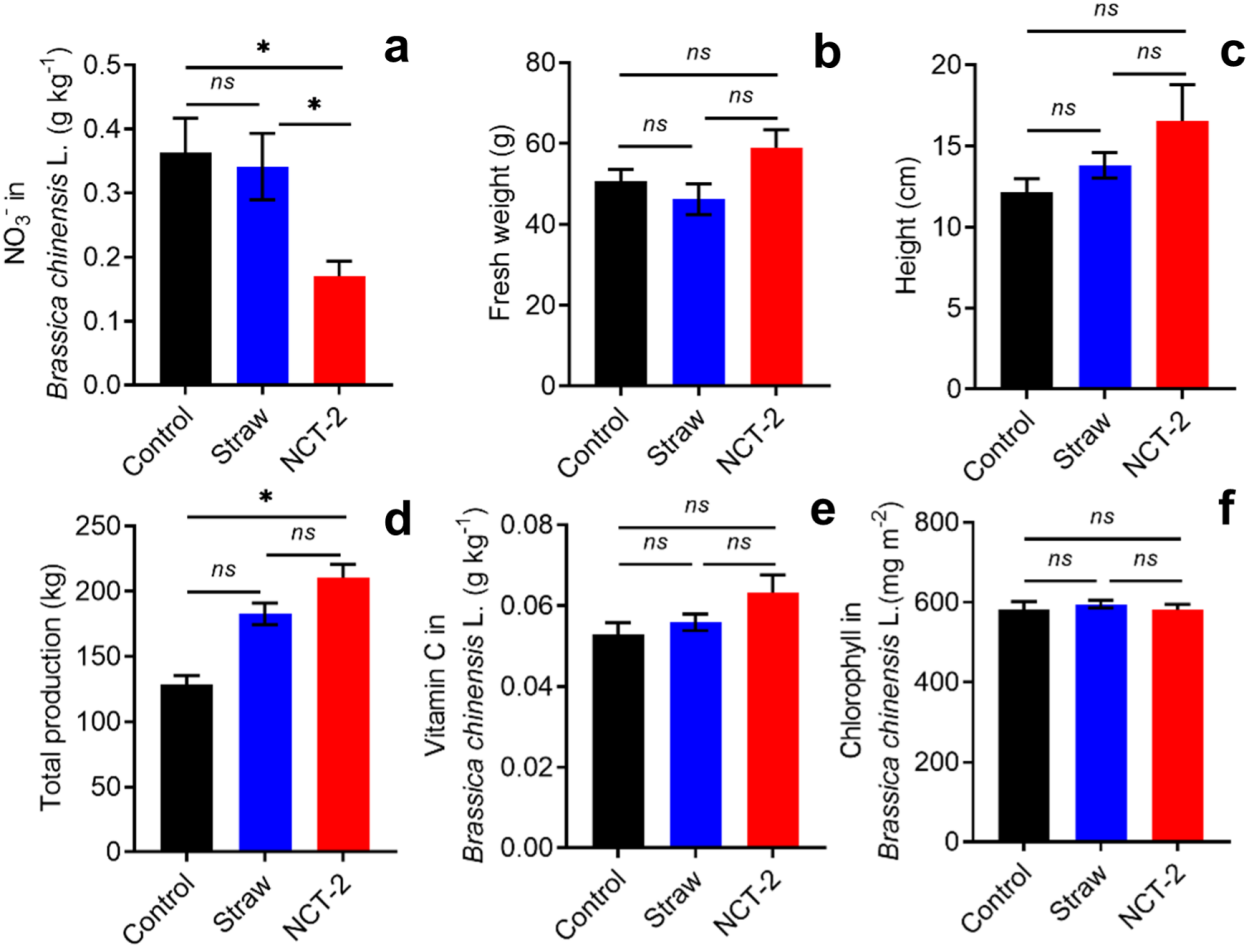
Nitrate accumulation and physiological properties of B. chinensis in NCT and straw treatment groups at the end of the experiment. (A) Nitrate concentration, (B) fresh weight, (C) height, (D) total production, (E) Vc concentration, (F) chlorophyll concentration. The asterisk (*) indicates a significant difference (*p* < 0.05). The *ns* means no significant.

As shown in Fig. 3A, NCT-2 inoculation significantly reduced the nitrate concentration in *B. chinensis*. Although the fresh weight, height, and total production of *B. chinensis* were higher in the NCT treatment group (Figs. 3B–3D), these differences were not significant. For this reason, the significant reduction of *B. chinensis* nitrate concentration was not a consequence of plant growth promotion by the NCT-2 inoculant. In addition, NCT treatment resulted in a higher Vc concentration in *B. chinensis* than straw treatment (Fig. 3E), while chlorophyll concentration was similar between treatment groups (Fig. 3F).

### Effect of NCT-2 inoculation on microbial succession in secondary salinized soil

**Figure 4:**
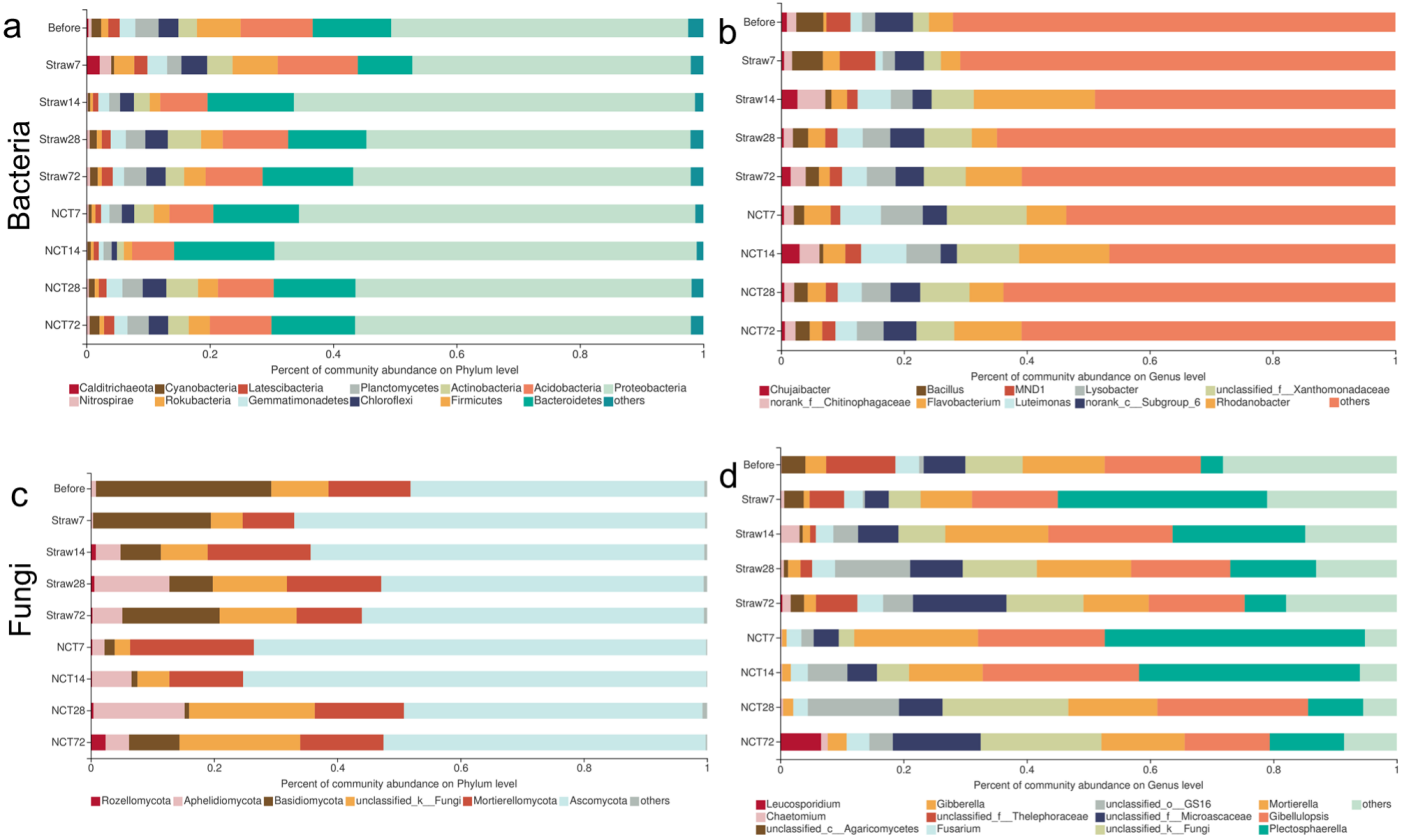
Microbial community succession following NCT and straw treatments. (A) Bacterial community abundance at the phylum level, (B) bacterial community abundance at the genus level, (C) fungal community abundance at the phylum level, (D) fungal community abundance at the genus level.

A total of 1,378,679 and 1,134,954 high-quality 16S rRNA and ITS gene amplicon sequences were generated by Illumina MiSeq sequencing after sequence processing, with an average length of 396-bp and 244-bp respectively (Tables S1 and S2). Then, the 16S rRNA and ITS gene sequences were clustered into 1,879 and 392 OTUs at 97% similarity for all samples. It is reported that high-coverage dataset from sustained sufficient sequencing depth could capture the majority of the known bacterial taxa and functional groups present in the samples (Mas-Lloret et al., 2020). In our present study, the coverages of all samples were more than 99% (Tables S3 and S4). It might characterize almost the entire microbial community. High-throughput sequencing revealed that the predominant bacterial phyla in soils at all stages in both treatment groups were Proteobacteria, Bacteroidetes, and Acidobacteria, accounting for over 70% of the total bacterial sequences (Fig. 4A). On the 7th day, the bacterial community in the straw treatment group had a similar structure to that in soil before the experiment (Figs. S1 and S2). The relative abundance of Proteobacteria exhibited an obvious increase following NCT treatment (Fig. S1). Likewise, when compared to the straw treatment group, NCT-2 induced more obvious changes at the genus level on the 7th day (Fig. 4B). Nonetheless, from the 28th day until the 72nd day, both treatment groups had similar bacterial community structure at both the phylum or genus level.

The predominant fungal phylum was Ascomycota, which accounted for over 40% of the total fungal sequences per soil sample (Fig. 4C). Compared with straw treatment, NCT-2 inoculation had a strong inhibitory effect on Basidiomycota and promoted Mortierellomycota on the 7th day (Fig. 4C). In contrast to the soil bacterial community, the addition of straw powder considerably changed fungal community structure at the genus level on the 7th day (Fig. 4D). It is noteworthy that the unclassified genera belonging to Thelephoraceae family and Agaricomycetes class disappeared in NCT treatment from the 7th day (Fig. 4D). While the relative abundance of *Leucosporidium* in NCT treatment was about 20 times higher than that in straw treatment on the 72nd day (Fig. 4D). At the end of the experiment, both treatment groups showed distinct fungal community structure, whether at the phylum or genus level (Figs. S3 and S4).

Overall, greater change was observed in the fungal community relative to the bacterial community following NCT-2 inoculation. Of note, bacterial community structures did shift, yet to a considerably lesser degree. Indigenous bacterial communities were highly resistant against the introduction of NCT-2. These two treatment groups exhibited similar bacterial community structure but distinct fungal community structure.

### Effect of NCT-2 inoculation on the microbial diversity of secondary salinized soil

With regard to soil bacterial diversity, NCT-2 inoculation had no significant effect on the bacterial Shannon, Simpson, Ace, and Chao indexes when compared to straw treatment (Table S3), which implied that bacterial diversity was not affected by NCT-2. With respect to soil fungal diversity, there was no significant difference in the Simpson index between the two treatments (Table S4). However, Ace index values on the 7th, 14th, and 28th day were significantly lower in the NCT treatment compared to straw treatment. Further, the NCT-2 inoculant significantly reduced the fungal Shannon index on the 14th day as well as Chao index values on the 7th and 14th day. These results indicated that fungal communities were more sensitive to NCT-2 inoculation than bacterial communities.

**Figure 5:**
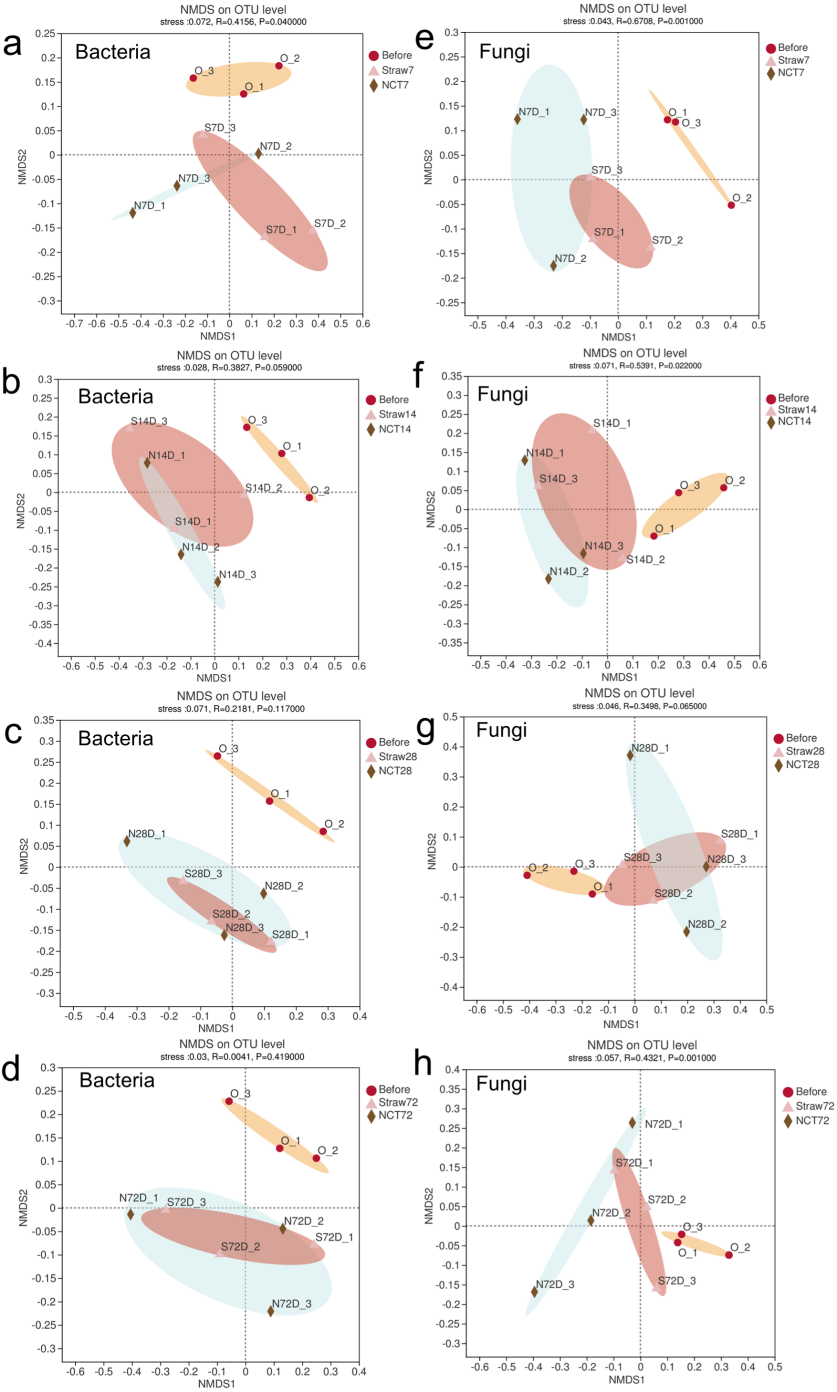
Non-metric multidimensional scaling (NMDS) plots of microbial compositions following NCT and straws treatment. (A–D) NMDS plots based on OTU distribution showed the soil bacterial compositions on the 7th (A), 14th (B), 28th (C), 72th (D) day, (E–H) NMDS plot based on OTU distribution showed the soil fungal on the 7th (E), 14th (F), 28th (G), 72th (H) day.

NMDS analysis revealed that bacterial populations were similar except apart from the bacterial community on the 7th day, while fungal populations differed between NCT-2 and straw treatment soil at the later stage (Fig. 5). It is suggested that the NCT-2 inoculant had a significant effect on soil bacterial composition at the early stage, but its effect was negligible in the late stage. Fungal composition was considerably altered following NCT-2 inoculation.

### Correlation among soil chemical properties and microbial community structure

**Figure 6:**
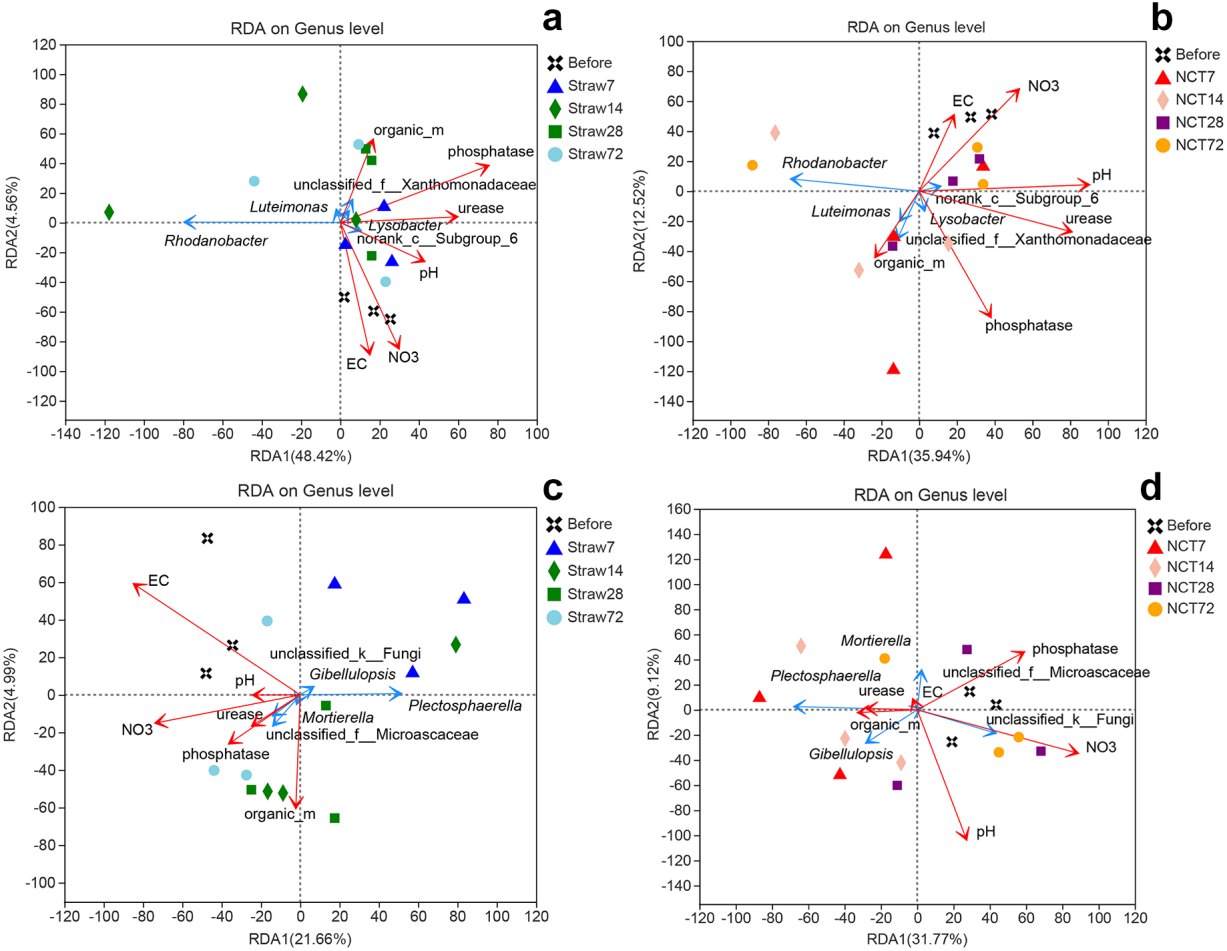
Redundancy analysis (RDA) deciphering the relationships among microbial taxa (bacterial and fungal) and soil characteristics. (A) The relationships between bacteria in straw-treated soil and soil characteristics, (B) The relationships between bacteria in NCT-treated soil and soil characteristics, (C) The relationships between fungi in straw-treated soil and soil characteristics, (D) The relationships between fungi in NCT-treated soil and soil characteristics. The values of axes are the percentages explained by the corresponding axis. The abbreviation of soil characteristics: electrical conductivity (EC), nitrate content (NO3), Urease activity (Urease), phosphatase activity (phosphatase), organic matter content (organic_m).

The correlation between the microbial structure and environmental factors was established by Spearman correlation analysis and redundancy analysis (RDA). The Spearman correlation coefficients and the explained variations of RDA were shown in Table S5. Of the variation, 52.98% and 48.46% were explained by both RDA1 and RDA2 axes in bacterial community structures under straw and NCT treatment respectively (Figs. 6A and 6B). Both RDA1 and RDA2 axes explained 26.65% and 40.89% in fungal community structures under straw and NCT treatment, respectively (Figs. 6C and 6D). Among the six soil chemical properties, EC and nitrate content were positively correlated with the bacterial and fungal community in soils before both treatments. The pH, urease activity, phosphatase activity, and organic matter content explained the total shift in bacterial communities resulting from NCT and straw treatment (Figs. 6A and 6B). On the 7th day, all the six soil chemical properties were negatively correlated with fungal community in straw treatment (Fig. 6C), however, the organic matter content and urease activity were positively correlated with fungal community in NCT treatment (Fig. 6D). The correlation between fungal community and soil properties showed convergence in both treatments over time. The fungal communities on the 72nd day and before treatment showed similar correlations with the soil properties (Figs. 6C and 6D). *Rhodanobacter* was positively associated with organic matter content and negatively associated with five other factors in NCT-treated soil (Fig. 6A). Further, this genus was negatively associated with all six environmental factors in straw-treated soil (Fig. 6B). For fungi, *Plectosphaerella* was positively associated with urease activity, while being negatively associated with phosphatase activity, pH, and nitrate content in NCT-treated soil (Fig. 6C). However, *Plectosphaerella* was negatively related to all six environmental factors in straw-treated soil (Fig. 6D). These results suggested that *Rhodanobacter* and *Plectosphaerella* are sensitive to changes in environmental factors.

### Effect of NCT-2 inoculation on microbial functions

PICRUSt and FUNGuild were used for functional analysis of soil bacteria and fungi. At time points, the bacteria in both treatment groups were enriched in functions including metabolism, genetic information processing, environmental information processing, cellular processes, human diseases, and organismal systems (Fig. S5). Among predicted KEGG pathways, metabolism was the most abundant category, followed by environmental information processing. However, these enriched pathways were similar between all samples, indicating that the bacterial functions were more stable than structure.

**Figure 7:**
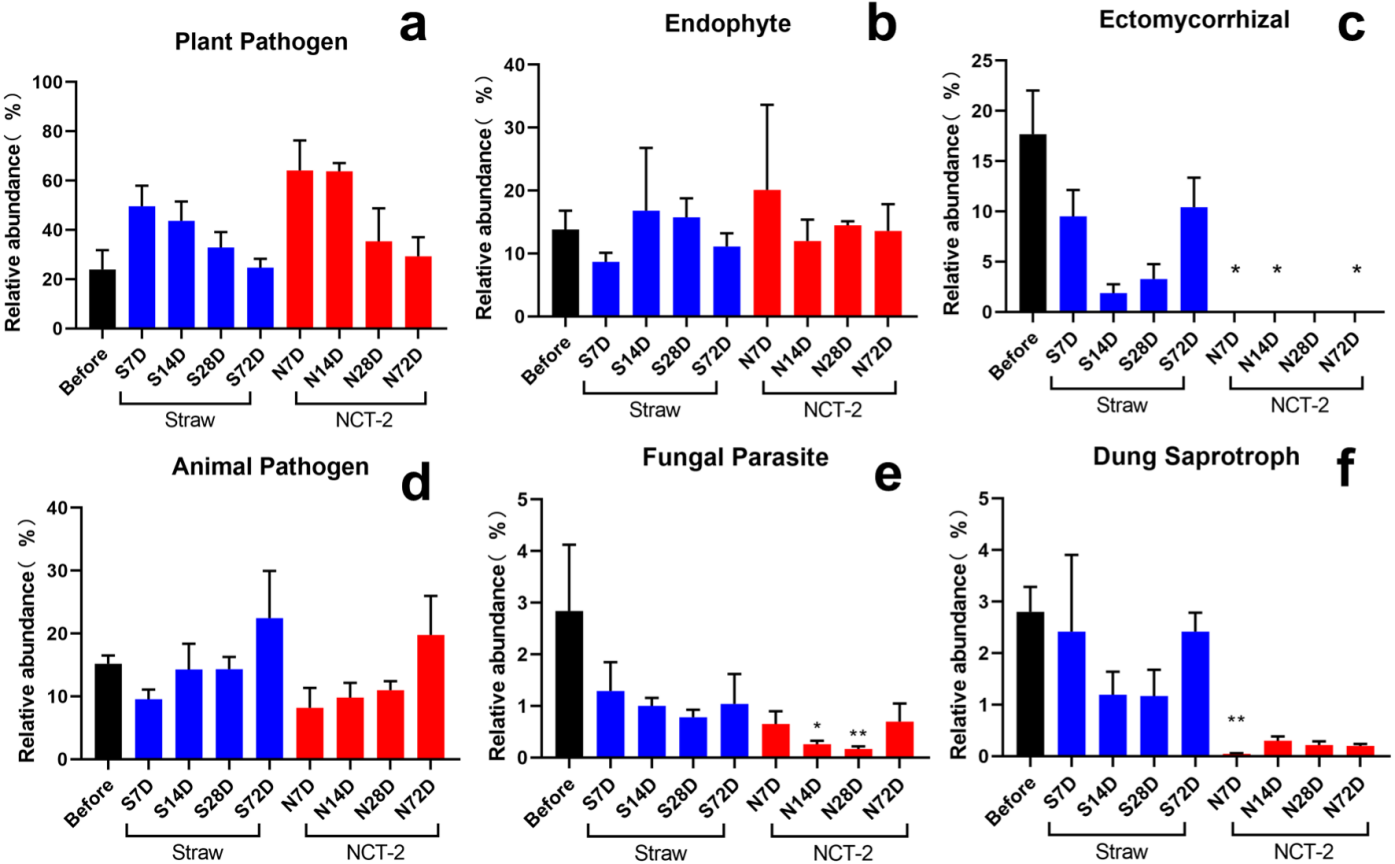
The relative abundance of fungal functional groups (A-F) in NCT- and straw-treated soil. The asterisk indicates a significant difference with before treatment (*, *p* < 0.05; **, *p* < 0.01).

FUNGuild, a tool for analyzing fungal trophic modes, has previously been applied in determining the functional roles of fungi (Nguyen et al., 2016). Grouping of taxa into functional guilds could reveal niche differentiation and specific ecosystem functions of the fungal community (Chen, Wang & Wen, 2018). Compared to bacteria, fungal functions showed larger differences between various samples (Fig. S6). Especially from the 7th day until the 72nd day, ectomycorrhizal fungi were completely suppressed in the NCT treatment, suggesting the strong inhibitory effect of the NCT-2 inoculant (Fig. 7C). Moreover, the NCT treatment also showed inhibition effects on fungal parasite and dung saprotroph (Figs. 7E and 7F).

## Discussion

In the present study, the nitrate content in soil showed insignificant difference between straw treatment and NCT treatment, although NCT-2 inoculant significantly promoted the nitrate removal from soil in our previous study (Chu et al., 2018). It may be due to the higher background value of nitrate content in the present study (0.71 g kg^−1^) than that in our previous study (0.097 g kg^−1^ ∼0.54 g kg^−1^). In consideration of the smaller biomass and the shorter growth period of *B. chinensis* in the present study than maize in the previous study, the NCT-2 inoculant dosage was lower in the present study (below 6  × 10^4^ CFU g^−1^ soil) than that in our previous study (2  × 10^5^ CFU g^−1^). So, it is hard to perform a reasonable comparison. The insignificant nitrate removal effect of NCT-2 inoculant may be ascribed to the usage of low dose of NCT-2 inoculant in soil with high nitrate content. It indicated that enough NCT-2 should be inoculated into severe secondary salinized soil in order to get satisfied amendment effect in practical application. Moreover, the same protocol as the previous study will be used to get a meaningful comparison in the further study.

The nitrate content of soil in both treatments decreased in the early stage of the experiment, suggesting that *B. chinensis* took advantage of soil nitrate for their growth. However, the nitrate content increased again in the later stage in straw treatment, which may be related to the low biomass and short growth season of *B. chinensis*, as well as the nitrogen transformation. In contrast, the increase of soil nitrate in NCT treatment was inhibited, and the nitrate content in soil was below 0.5 g kg^−1^ throughout the experiment. Because the growth of *B. chinensis* showed no significant difference between two treatments, it can be supposed that NCT-2 inoculant promoted the soil nitrate reduction by influencing the soil microbial population and their nitrogen metabolism pathways. In addition, the significantly low nitrate content in *B. chinensis* in NCT treatment was likely related to the lower nitrate content in soil.

Our hypothesis was that the amendment effect of NCT-2 inoculant on secondary salinized soil is ascribable to the shift of soil microbial community. However, our results indicated that the bacterial communities in both treatments were just distinct at the beginning of the experiment (on the 7th day). The relative abundance of Proteobacteria and *Flavobacterium* showed exhibited obvious increase following NCT treatment on the phylum and genus level, respectively. The *α*, *β*, and *γ* Proteobacteria possess the ability to obtain energy from dissimilatory reduction of nitrate into nitrite (Philippot et al., 2002). Many members of the genus *Flavobacterium* reportedly reduce nitrate to nitrogen (Kim et al., 2012). Hence Proteobacteria and *Flavobacterium* may help reduce the nitrate in NCT-treated soil. Nevertheless, the original differences in bacterial community were not visible at the end of the experiment. By contrast, the fungal communities also showed the convergence effect, however, the composition were still obviously different after 72 days. The NCT treatment numerically increased the relative abundance of the phylum Mortierellomycota and the genus *Leucosporidium*. It is reported that Mortierellomycota plays a vital role in nitrogen cycling in soil-plant systems (Dang et al., 2020). And the genus *Leucosporidium* has been reported to be able to assimilate nitrate (García-Lugo et al., 2000). Therefore, Mortierellomycota and *Leucosporidium* were conducive to nitrate reduction in NCT-treated soil. The convergence of soil microbial community may be related to the strong selective pressures of plants, which has been reported in other studies (Yergeau et al., 2015). The NCT treatment decreased the pH value in soil, and it seemed that the relative abundances of Proteobacteria, *Flavobacterium*, Mortierellomycota and *Leucosporidium* were negatively related to soil pH in the present study. However, only the Proteobacteria phylum was reported to be negatively related to pH (Ganz et al., 2012), while *Flavobacterium* (Zhalnina et al., 2015), Mortierellomycota (Ding et al., 2021; Shi et al., 2020) and *Leucosporidium* (Dreesens, Lee & Cary, 2014) were reported to be positively related to pH. Hence the divergent results need further study. Although some studies have found that short-term shifts in microbial communities had lasting effects (Yergeau et al., 2015), similar function prediction of bacterial communities in all samples suggested that final nitrate reduction in soil and in *B. chinensis* would be unrelated to early changes in bacterial communities. While the amendment effect should have more to do with the changes of fungal communities based on the differences in structure and function at the end of the experiment. Consequently, our hypothesis was partly supported. Instead of modifying the whole microbial communities, NCT-2 inoculant amended the secondary salinized soil by shifting the fungal communities.

Although transient shift of bacterial community structure after inoculation with NCT-2 was observed, the bacterial function remained stable throughout. Thus, changes in bacterial community structure may not influence function due to bacterial redundancy, as different bacterial species may carry out the same functions (Kennedy, 1999; Nannipieri et al., 2003). Other studies have also reported minor changes in the diversity of indigenous bacterial communities, including *Bacillus* species, following bacterial inoculation (Ambrosini, de Souza & Passaglia, 2016; Björklöf, Sen & Jørgensen, 2003; Chowdhury et al., 2013; Felici et al., 2008; Trabelsi & Mhamdi, 2013). Some researchers proposed that the low disturbance of microbial systems is linked to plant growth promotion (Ramos et al., 2003). Hence, the effect of NCT-2 inoculant for secondary salinized soil remediation may be related to low disturbance of the indigenous soil bacterial community, in addition to other mechanisms such as its high nitrate assimilation capacity (Chu et al., 2017). However, both structure and function of fungal community were more sensitive to NCT-2 inoculation than bacterial community. The different responses to NCT-2 inoculation between fungal and bacterial communities could be owing to the more intimate nature of the relationship between fungi and plants (Yergeau et al., 2015). Moreover, in the soil environment, bacteria and fungi can be antagonists (Schrey et al., 2012), and their competition has been proved to affect the microbiome (Meidute, Demoling & Bååth, 2008). Therefore, one possible explanation for greater changes of fungal community could be that inoculation with NCT-2 brought a disproportionate direct effect on fungi. Previous studies reported similar results, suggesting that some bacterial inoculants, including *Bacillus*, influenced fungal growth (Bonfante & Anca, 2009; Probanza et al., 2001; Toljander et al., 2006).

Interestingly, ectomycorrhizal fungi were detected in greenhouse soil even though their abundance was low. Ectomycorrhizas are found on woody plants ranging from shrubs to forest trees (Reddy & Satyanarayana, 2006), while Brassicaceae are nonmycorrhizal (Zeng, Mallik & Setliff, 2003). However, it has been reported that some species of Brassicaceae significantly simulated hyphal growth of ectomycorrhizal fungi by producing one or more growth-stimulating compounds (Zeng, Mallik & Setliff, 2003). Other researchers suggested that ectomycorrhizal fungi are saprotrophic organisms (Martin et al., 2008). Moreover, the application of organic fertilizers was reported to promote the relative abundance of ectomycorrhizal fungi (Wang et al., 2017). These observations may explain the presence of ectomycorrhizal fungi in the present study. Although some strains of *Bacillus* have been identified as ‘mycorrhizal helper bacteria’ (MHB) (Frey-Klett, Garbaye & Tarkka, 2007), the NCT-2 inoculant exhibited a strong inhibitory effect on ectomycorrhizal fungi from the 7th day until the 72nd day. Two severely inhibited genera belonging to Thelephoraceae family and Agaricomycetes class were both members of *Agaricomycetes*, which contains the vast majority of ectomycorrhizae-forming fungi (Hibbett & Matheny, 2009). A variety of genera have been reported to inhibit the growth of ectomycorrhizal fungi, including *Bacillus* (Bending et al., 2002; Chet et al., 1990; De Boer et al., 2001; Reddy & Satyanarayana, 2006; Varese et al., 1996). Bacterial inhibition of ectomycorrhiza could be caused by: (1) micro-scale nutrient and water competition in the rhizosphere (Germida & Walley, 1996; Shishido & Chanway, 1998); (2) bacterial niche exclusion at roots (Domenech et al., 2004); and/or (3) the production of anti-fungal metabolites by bacteria (Barea et al., 1998). It has been observed that the growth of crop plants may be inhibited when they are infected by mycorrhizal fungi while growing in fertile soil (Francis & Read, 1995). Hence, NCT-2 inoculant-mediated inhibition of ectomycorrhizal fungi may be beneficial for crop plant cultivation. Although the majority of ectomycorrhizal fungi are reported to prefer ammonium as an inorganic nitrogen source, the capacity to grow on nitrate is also widely distributed among them (Nygren et al., 2008) and some ectomycorrhizal species even prefer nitrate over ammonium (Kemppainen & Pardo, 2013; Scheromm, Plassard & Salsac, 1990). Therefore, it can be speculated that the ectomycorrhizal species existed in soil in this experiment might prefer nitrate as their nitrogen source, and the inhibited ectomycorrhizal species might be related to the low nitrate content in the NCT-treated soil. Moreover, fungal parasite and dung saprotroph were incompletely suppressed in the NCT treatment. *Bacillus* strains treated with coated nanoparticles was reported to inhibit the growth of harmful fungal parasite within rhizosphere (Gouda et al., 2018). The inhibition effects can be due to their ability to induce host-plant’s natural defense response mechanisms (Lastochkina et al., 2018). The growth of dung saprotroph requires a great deal of nutrients, including nitrogen sources (Han, Dong & Zhang, 2021). The lower soil nitrate content in the NCT treatment may be the reason of the inhibition effect on dung saprotroph.

*B. megaterium* NCT-2 is a member of the *Bacillus* genus and Firmicutes phylum, however, the relative abundances of *Bacillus* and Firmicutes in soil were still low following the inoculation with NCT-2. Our previous study found that NCT-2 population drastically dropped at the beginning of inoculation, and then fluctuated (Chu et al., 2018). On the other hand, as an endophyte (Chu et al., 2018), a part of NCT-2 inoculants may colonize on plants. Hence inoculation with NCT-2 may be not helpful to increase the relative abundance of *Bacillus* and Firmicutes in soil, but will result in the fluctuation of microbial community. As shown in the present study, NCT-2 inoculant did modify the microbial community, especially fungal community. Moreover, NCT-2 inoculant significantly promoted the growth of maize in our previous study (Chu et al., 2018), however, it showed no significant effect on the growth of *B. chinensis* in the present study. One possible explanation could be that the colonization of NCT-2 on plants is selective, so that NCT-2 may have a weaker colonization ability on *B. chinensis* than on maize to play the plant growth promotion effects. Therefore, it still needs to verify the presence of the strain NCT-2 in endophyte community based on the present data. In future studies, the colonization pattern and dynamics of NCT-2 in secondary salinized soil and various plants will be carried out.

In the present study, the archaeal community in soil was not profiled. The archaeal community makes up only about 2% of all 16S rRNA gene sequences across all soils (Bates et al., 2011). Although there are numerous lines of evidence suggesting that archaea may function as important soil nitrifiers, more and more studies have produced divergent results (Bates et al., 2011). Especially in agricultural soil, bacteria rather than archaea dominate nitrification activity (Banning et al., 2015; Jia & Conrad, 2009). The studies involved in the dynamics of archaeal community in secondary salinized soil are scarce. In the further study, the effect of NCT-2 inoculant on archaeal community and the soil control for 16S rRNA/ITS gene amplicon sequencing will be investigated.

The limitations of sequencing the conserved 16S rRNA gene are that the annotation is based on putative association of the 16S rRNA gene with a taxa defined as an operational taxonomic unit (OTU). In general, OTUs are analyzed at the phyla or genera level, and can be less precise at the species level. An alternative approach is whole genome shotgun sequencing (WGS) which uses sequencing with random primers to sequence overlapping regions of a genome. The major advantages of the WGS method are that the taxa can be more accurately defined at the species level. However, WGS is more expensive and requires more extensive data analysis. In the present study, the 16S rRNA amplicon method was used to investigate the time course of taxonomically profiling microbial communities in soil at genus level in response to strain NCT-2 inoculation. Unlike metagenomic approaches, 16S rRNA amplicon method does not provide direct evidence of a community’s functional capabilities, such as the functional genes that are present in the genomes of community members. Moreover, predicting microbiome function from taxonomic composition is difficult, as microbiota composition was found not to mirror functionality (Vallès et al., 2014). There is a large amount of horizontal gene transfer which is mediated by phage or plasmid transfer in bacteria. The movement of genes among genomes *via* horizontal gene transfer can blur many feature of microbial identity and community composition, as function and species identity can become decoupled (Burke et al., 2011). The PICRUSt tool can predict functional category abundances based on an input marker gene. However, PICRUSt analysis for bacterial function prediction from 16S sequence relies in part on phylogeny and species identities, and is likely to be less informative (Koskella, Hall & Metcalf, 2017). Thus, the bacterial functions discussed in the present study may not exactly be from the microorganisms as reported in previous studies. And this was another limitation of the methods used in this study. In addition, FUNGuild is a tool that can be used to taxonomically parse fungal OTUs by ecological guild independent of sequencing platform or analysis pipeline, connecting the taxonomic identification to trophic guilds. It has been used to predict the nutritional and functional groups of the fungal communities. Due to the limitations of 16S rRNA amplicon method in the current study, the WGS method will be used to investigate the functional genes, microbial pathways with encouraging prospects for future study. Moreover, the abundance of functional genes with rare horizontal gene transfer events will also be measured to characterize bacterial functions for future study.

Our results indicated that *B. megaterium* NCT-2 can inhibit the rebound of soil nitrate instead of Cl^−^ and K^+^ in the late stage of the experiment, which was expected, as *B. megaterium* NCT-2 had been originally isolated from secondary salinized soil with high nitrate content (Shi et al., 2014). Typically, the ecological adaptability refers to the biological characteristics and the ecological conditions of the local environment (Pu et al., 2021). Successful species for restoration must be able to survive and propagate themselves across a particular region; thus they must have certain ecological adaptability (Zhang, De Angelis & Zhuang, 2011). The ecological adaptability of NCT-2 makes it a good candidate for use in secondary salinized soil amendment. Up to 80% of the nitrate in human diets is provided by vegetables (Lairon, 2010; Magkos, Arvaniti & Zampelas, 2006). Nitrate can be easily transformed into nitrite, which binds certain amines and amides to produce nitrosamines with carcinogenic potential (Lairon, 2010; Magkos, Arvaniti & Zampelas, 2006). Therefore, nitrate has been associated with cancer risk (Garcia & Teixeira, 2017). The effect of NCT-2 inoculant on nitrate reduction in *B. chinensis* suggested its potential for application in vegetable cultivation in secondary salinized soil.

## Conclusions

The *B. megaterium* NCT-2 inoculant can significantly reduce nitrate content in *B. chinensis*. Although the reduction of soil nitrate by inoculation with NCT-2 was insignificant, NCT-2 inoculant inhibited the increase of soil nitrate in the later stage of the experiment. The structure and function of the soil fungal community were subject to greater changes than those observed for bacteria in response to NCT-2 inoculation. Interestingly, the NCT-2 inoculant completely suppressed the relative abundance of ectomycorrhizal. At the end of the experiment, soils from both treatment groups had similar bacterial structure and function, but distinct fungal structure and function. Hence, the remediation effect of NCT-2 inoculant should be related to the shifts of fungal community in soil. The present study advances the current understanding of microbial responses to bacterial inoculant treatment, contributing to a more efficient and reliable use of bacterial inoculants for secondary salinized soil remediation.

## Supplemental Information

10.7717/peerj.12309/supp-1 Supplemental Information 1 Valid sequences of each sample Table S1. Valid 16S rDNA sequences of each sample; Table S2. Valid ITS sequences of each sample

10.7717/peerj.12309/supp-2 Supplemental Information 2 Alpha diversity indexes in NCT and straws treatments Table S3 Alpha diversity indexes of bacterial communities in NCT and straws treatments. Table S4 Alpha diversity indexes of fungal communities in NCT and straws treatments.

10.7717/peerj.12309/supp-3 Supplemental Information 3 Table S5. Correlation between soil chemical properties and microbial structure

10.7717/peerj.12309/supp-4 Supplemental Information 4 Bacterial abundance at phylum levels

10.7717/peerj.12309/supp-5 Supplemental Information 5 Bacterial abundance at genus levels

10.7717/peerj.12309/supp-6 Supplemental Information 6 Fungal abundance at phylum levels

10.7717/peerj.12309/supp-7 Supplemental Information 7 Fungal abundance at genus levels

10.7717/peerj.12309/supp-8 Supplemental Information 8 Bacterial functional profiles

10.7717/peerj.12309/supp-9 Supplemental Information 9 Fungal functional profiles

10.7717/peerj.12309/supp-10 Supplemental Information 10 Raw data for Figs. 1–3

10.7717/peerj.12309/supp-11 Supplemental Information 11 OTUs of 16S rDNA

10.7717/peerj.12309/supp-12 Supplemental Information 12 OTUs of ITS

## Additional Information and Declarations

### Data Availability

The following information was supplied regarding data availability: All sequencing data generated in this study are available at NCBI Sequence Read Archive (SRA): PRJNA704178.
