# Supplementary material for: Changes in soil bacterial and fungal communities in response to Bacillus megaterium NCT-2 inoculation in secondary salinized soil
Source: PeerJ. 2021 Oct 12;9:e12309. doi: 10.7717/peerj.12309 (PMC8519178; doi:10.7717/peerj.12309)
Supplement: Supplemental Information 1 — Table S1. Valid 16S rDNA sequences of each sample; Table S2. Valid ITS sequences of each sample [file peerj-09-12309-s001.docx]

**Table S1. Valid 16S rDNA sequences of each sample**

| Sample | Seq_num | Base_num | Mean_length | Min_length | Max_length |
| --- | --- | --- | --- | --- | --- |
| Before_1 | 57423 | 22745961 | 396.1124 | 251 | 426 |
| Before_2 | 50581 | 20039887 | 396.194 | 324 | 432 |
| Before_3 | 42653 | 16887693 | 395.9321 | 326 | 423 |
| N7D_1 | 54348 | 21532812 | 396.2025 | 279 | 450 |
| N7D_2 | 43753 | 17325936 | 395.9942 | 267 | 436 |
| N7D_3 | 58958 | 23347371 | 396.0001 | 317 | 432 |
| N14D_1 | 54204 | 21472954 | 396.1507 | 356 | 449 |
| N14D_2 | 56669 | 22439412 | 395.9733 | 201 | 456 |
| N14D_3 | 46890 | 18562135 | 395.8655 | 364 | 432 |
| N28D_1 | 47128 | 18664175 | 396.0316 | 232 | 418 |
| N28D_2 | 48577 | 19244813 | 396.1713 | 322 | 432 |
| N28D_3 | 50934 | 20177572 | 396.1513 | 364 | 454 |
| N72D_1 | 43993 | 17433970 | 396.2896 | 284 | 425 |
| N72D_2 | 51853 | 20540028 | 396.1203 | 311 | 434 |
| N72D_3 | 56346 | 22306823 | 395.8901 | 353 | 424 |
| S7D_1 | 59596 | 23608062 | 396.135 | 235 | 460 |
| S7D_2 | 47566 | 18848217 | 396.254 | 360 | 432 |
| S7D_3 | 51170 | 20267003 | 396.072 | 359 | 451 |
| S14D_1 | 44429 | 17593476 | 395.9908 | 385 | 432 |
| S14D_2 | 43195 | 17106464 | 396.0288 | 252 | 455 |
| S14D_3 | 58782 | 23290372 | 396.2161 | 335 | 420 |
| S28D_1 | 59970 | 23758634 | 396.1753 | 320 | 449 |
| S28D_2 | 48369 | 19160427 | 396.1303 | 251 | 421 |
| S28D_3 | 59089 | 23403931 | 396.0793 | 301 | 433 |
| S72D_1 | 48734 | 19304614 | 396.1221 | 249 | 431 |
| S72D_2 | 44264 | 17520676 | 395.8222 | 306 | 435 |
| S72D_3 | 49205 | 19493092 | 396.1608 | 348 | 433 |

**Table S2. Valid ITS sequences of each sample**

| Sample | Seq_num | Base_num | Mean_length | Min_length | Max_length |
| --- | --- | --- | --- | --- | --- |
| Before_1 | 51727 | 12930542 | 249.9766 | 202 | 355 |
| Before_2 | 58738 | 15019810 | 255.7086 | 200 | 340 |
| Before_3 | 48892 | 12392677 | 253.4704 | 200 | 346 |
| N7D_1 | 18826 | 4550025 | 241.6884 | 200 | 331 |
| N7D_2 | 39011 | 9239849 | 236.8524 | 201 | 352 |
| N7D_3 | 37342 | 8907800 | 238.5464 | 201 | 326 |
| N14D_1 | 40687 | 9706312 | 238.5605 | 201 | 326 |
| N14D_2 | 42409 | 10106472 | 238.3096 | 201 | 354 |
| N14D_3 | 31230 | 7480034 | 239.5144 | 200 | 346 |
| N28D_1 | 28275 | 7074461 | 250.202 | 200 | 351 |
| N28D_2 | 39083 | 9335156 | 238.8546 | 200 | 353 |
| N28D_3 | 24822 | 5979987 | 240.9148 | 201 | 346 |
| N72D_1 | 32139 | 7842107 | 244.0059 | 201 | 345 |
| N72D_2 | 43246 | 10663629 | 246.5807 | 201 | 344 |
| N72D_3 | 32833 | 8043600 | 244.9852 | 200 | 356 |
| S7D_1 | 58698 | 14339755 | 244.2972 | 202 | 350 |
| S7D_2 | 45919 | 11460905 | 249.5896 | 200 | 349 |
| S7D_3 | 46312 | 11197937 | 241.7934 | 202 | 339 |
| S14D_1 | 43635 | 10583188 | 242.539 | 200 | 326 |
| S14D_2 | 53679 | 12954695 | 241.3364 | 202 | 333 |
| S14D_3 | 37814 | 9204924 | 243.4264 | 200 | 343 |
| S28D_1 | 39352 | 9242740 | 234.8734 | 201 | 354 |
| S28D_2 | 57405 | 13909784 | 242.3096 | 201 | 336 |
| S28D_3 | 43792 | 10750879 | 245.4987 | 201 | 355 |
| S72D_1 | 43793 | 10859721 | 247.9785 | 200 | 353 |
| S72D_2 | 43602 | 10922558 | 250.5059 | 202 | 344 |
| S72D_3 | 51693 | 12816370 | 247.9324 | 201 | 352 |
