# Supplementary material for: Changes in soil bacterial and fungal communities in response to Bacillus megaterium NCT-2 inoculation in secondary salinized soil
Source: PeerJ. 2021 Oct 12;9:e12309. doi: 10.7717/peerj.12309 (PMC8519178; doi:10.7717/peerj.12309)
Supplement: Supplemental Information 2 — Table S3 Alpha diversity indexes of bacterial communities in NCT and straws treatments. Table S4 Alpha diversity indexes of fungal communities in NCT and straws treatments. [file peerj-09-12309-s002.docx]

**Table S3 Alpha diversity indexes of bacterial communities in NCT and straws treatments**

| Diversity index | Day | Straw sample | NCT sample | *p* value |
| --- | --- | --- | --- | --- |
| Shannon | 0 | 6.01 ± 0.22 | |  |
|  | 7^th^ | 5.85 ± 0.09 | 5.22 ± 1.01 | 0.427 |
|  | 14^th^ | 4.90 ± 1.16 | 4.83 ± 0.56 | 0.949 |
|  | 28^th^ | 5.98 ± 0.35 | 5.82 ± 0.63 | 0.810 |
|  | 72^nd^ | 5.71 ± 0.68 | 5.64 ± 1.03 | 0.948 |
| Simpson | 0 | 0.01 ± 0.00 | |  |
|  | 7^th^ | 0.01 ± 0.01 | 0.03 ± 0.03 | 0.328 |
|  | 14^th^ | 0.06 ± 0.07 | 0.04 ± 0.03 | 0.790 |
|  | 28^th^ | 0.01 ± 0.01 | 0.01 ± 0.01 | 0.804 |
|  | 72^nd^ | 0.02 ± 0.02 | 0.03 ± 0.05 | 0.767 |
| Chao | 0 | 1709.36 ± 46.37 | |  |
|  | 7^th^ | 1646.90 ± 9.46 | 1602.64 ± 118.13 | 0.598 |
|  | 14^th^ | 1618.43 ± 170.81 | 1552.36 ± 106.94 | 0.715 |
|  | 28^th^ | 1755.37 ± 20.67 | 1702.32 ± 80.63 | 0.422 |
|  | 72^nd^ | 1708.93 ± 72.26 | 1703.79 ± 82.75 | 0.959 |
| Ace | 0 | 1700.28 ± 45.53 | |  |
|  | 7^th^ | 1627.72 ± 9.17 | 1612.79 ± 86.62 | 0.810 |
|  | 14^th^ | 1593.95 ± 161.41 | 1562.40 ± 105.69 | 0.850 |
|  | 28^th^ | 1749.09 ± 15.29 | 1701.43 ± 52.30 | 0.274 |
|  | 72^nd^ | 1703.79 ± 56.99 | 1696.34 ± 66.50 | 0.926 |
| Coverage | 0 | 0.991+0.001 | |  |
|  | 7^th^ | 0.99+0.000 | 0.99+0.002 | 0.777 |
|  | 14^th^ | 0.989+0.001 | 0.989+0.000 | 0.628 |
|  | 28^th^ | 0.992+0.001 | 0.991+0.001 | 0.816 |
|  | 72^nd^ | 0.991+0.001 | 0.991+0.001 | 0.924 |

**Table S4 Alpha diversity indexes of fungal communities in NCT and straws treatments**

| Diversity index | Day | Straw sample | NCT sample | *p* value |
| --- | --- | --- | --- | --- |
| Shannon | 0 | 3.54±0.10 | |  |
|  | 7^th^ | 2.90 ± 0.50 | 1.86 ± 0.32 | 0.161 |
|  | 14^th^ | 2.85 ± 0.13 | 2.05 ± 0.26 | 0.046 |
|  | 28^th^ | 2.95 ± 0.28 | 2.19 ± 0.11 | 0.050 |
|  | 72^nd^ | 3.35 ± 0.17 | 2.61 ± 0.23 | 0.053 |
| Simpson | 0 | 0.06±0.01 | |  |
|  | 7^th^ | 0.16 ± 0.08 | 0.31 ± 0.14 | 0.348 |
|  | 14^th^ | 0.16 ± 0.04 | 0.26 ± 0.08 | 0.241 |
|  | 28^th^ | 0.11 ± 0.02 | 0.23 ± 0.05 | 0.052 |
|  | 72^nd^ | 0.08 ± 0.01 | 0.13 ± 0.03 | 0.171 |
| Chao | 0 | 206.98±12.38 | |  |
|  | 7^th^ | 214.40 ± 18.26 | 150.95 ± 24.70 | 0.007 |
|  | 14^th^ | 227.06 ± 28.06 | 168.21 ± 15.60 | 0.017 |
|  | 28^th^ | 218.59 ± 22.65 | 163.80 ± 8.00 | 0.067 |
|  | 72^nd^ | 230.56 ± 29.80 | 169.13 ± 16.97 | 0.072 |
| Ace | 0 | 207.05±14.06 | |  |
|  | 7^th^ | 207.71 ± 22.73 | 146.87 ± 20.92 | < 0.01 |
|  | 14^th^ | 232.39 ± 32.07 | 165.40 ± 20.73 | 0.024 |
|  | 28^th^ | 219.58 ± 19.02 | 163.58 ± 8.45 | 0.038 |
|  | 72^nd^ | 225.72 ± 27.28 | 170.66 ± 23.40 | 0.094 |
| Coverage | 0 | 0.999+0.000 | |  |
|  | 7^th^ | 0.999±0.000 | 0.999±0.000 | 0.686 |
|  | 14^th^ | 0.998±0.000 | 0.998±0.000 | 0.5689 |
|  | 28^th^ | 0.998±0.000 | 0.999±0.000 | 0.278 |
|  | 72^nd^ | 0.999±0.000 | 0.999±0.000 | 0.412 |
