# Supplementary material for: Changes in soil bacterial and fungal communities in response to Bacillus megaterium NCT-2 inoculation in secondary salinized soil
Source: PeerJ. 2021 Oct 12;9:e12309. doi: 10.7717/peerj.12309 (PMC8519178; doi:10.7717/peerj.12309)
Supplement: Supplemental Information 3 [file peerj-09-12309-s003.docx]

**Table S5. Correlation between soil chemical properties and microbial structure**

|  |  | Bacteria | | | | Fungi | | | |
| --- | --- | --- | --- | --- | --- | --- | --- | --- | --- |
|  |  | RDA1 | RDA2 | r2 | *p*_values | RDA1 | RDA2 | r2 | *p*_values |
| Straw | phosphatase | 0.89 | 0.46 | 0.52 | 0.02 | -0.9 | -0.43 | 0.07 | 0.66 |
|  | NO_3_^-^ | 0.21 | -0.98 | 0.3 | 0.14 | -1 | 0.03 | 0.24 | 0.17 |
|  | urease | 0.97 | 0.26 | 0.23 | 0.22 | -0.92 | -0.39 | 0.03 | 0.83 |
|  | organic_m | 0.57 | 0.82 | 0.18 | 0.32 | 0.19 | -0.98 | 0.14 | 0.41 |
|  | pH | 0.98 | -0.22 | 0.11 | 0.49 | -0.98 | 0.19 | 0.02 | 0.86 |
|  | EC | -0.08 | -1 | 0.32 | 0.09 | -0.82 | 0.57 | 0.57 | 0.01 |
| NCT-2 | phosphatase | 0.61 | -0.79 | 0.38 | 0.07 | 0.8 | 0.6 | 0.31 | 0.1 |
|  | NO_3_^-^ | 0.71 | 0.7 | 0.24 | 0.19 | 0.99 | -0.16 | 0.39 | 0.05 |
|  | urease | 0.93 | -0.37 | 0.38 | 0.07 | -0.99 | -0.16 | 0.04 | 0.81 |
|  | organic_m | -0.49 | -0.87 | 0.08 | 0.62 | -0.97 | -0.24 | 0.05 | 0.73 |
|  | pH | 0.99 | -0.14 | 0.39 | 0.05 | 0.09 | -1 | 0.45 | 0.02 |
|  | EC | 0.28 | 0.96 | 0.09 | 0.56 | -0.11 | 0.99 | 0 | 0.99 |

electrical conductivity (EC), nitrate content (NO_3_^-^), Urease activity (urease), phosphatase activity (phosphatase), organic matter content (organic_m).
