# Supplementary figures and images for: Changes in soil bacterial and fungal communities in response to Bacillus megaterium NCT-2 inoculation in secondary salinized soil
Source: PeerJ. 2021 Oct 12;9:e12309. doi: 10.7717/peerj.12309 (PMC8519178; doi:10.7717/peerj.12309)

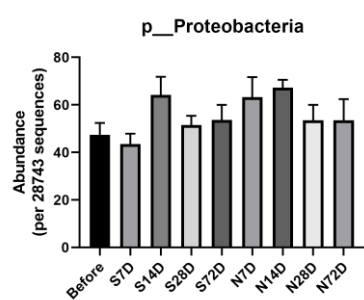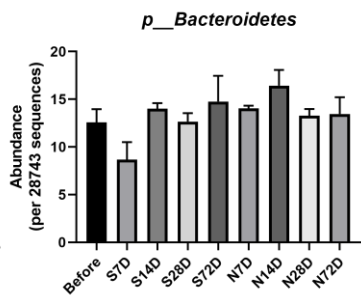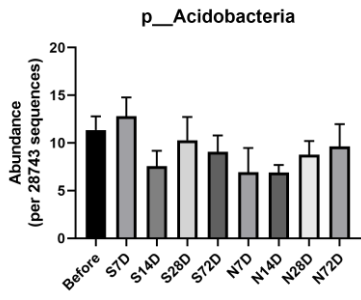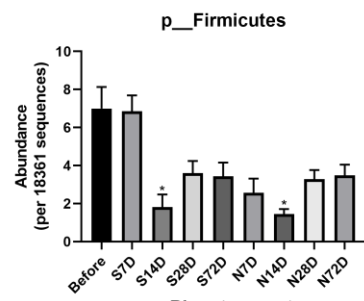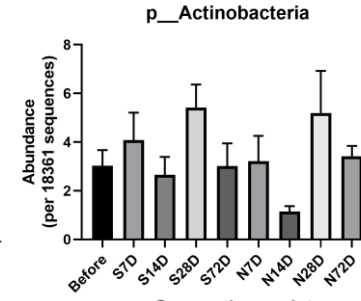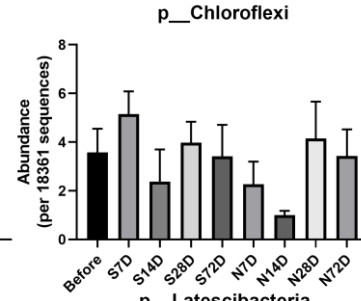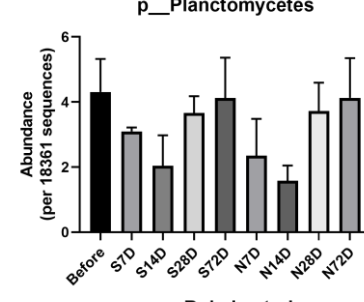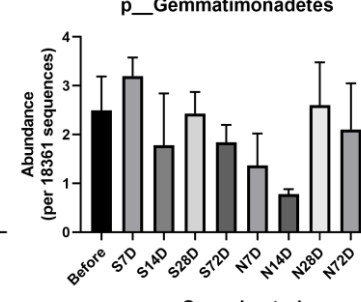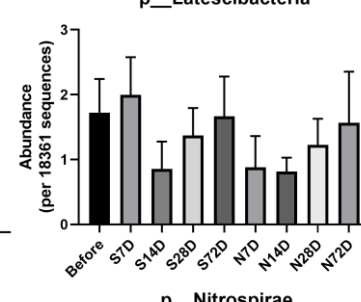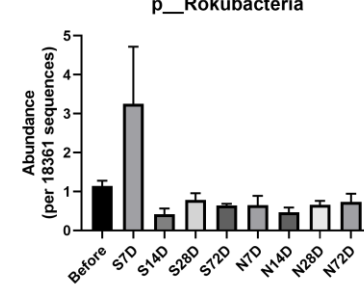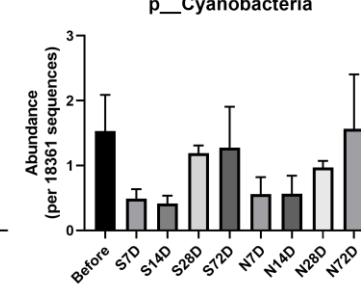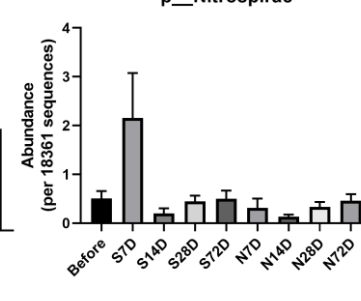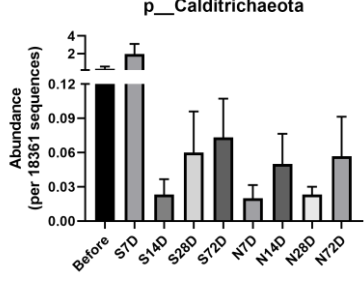

Supplement: Supplemental Information 4 [file peerj-09-12309-s004.pdf]

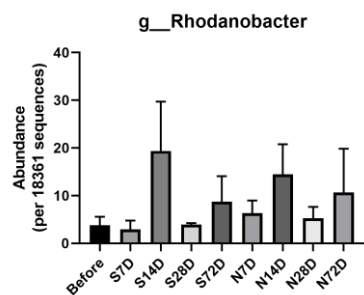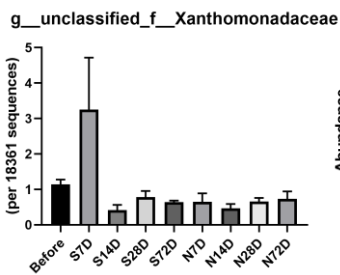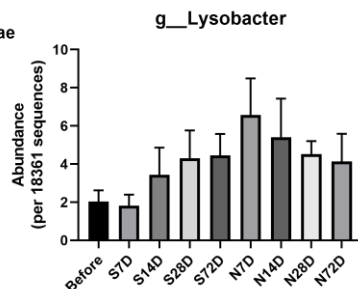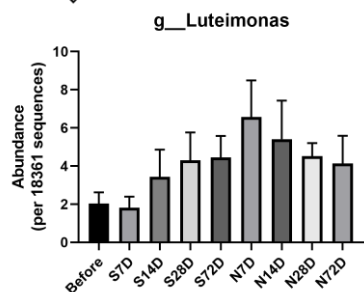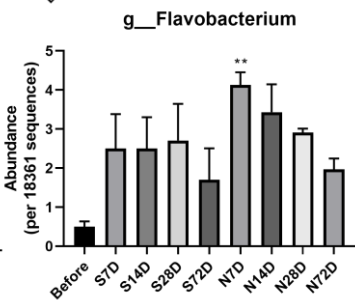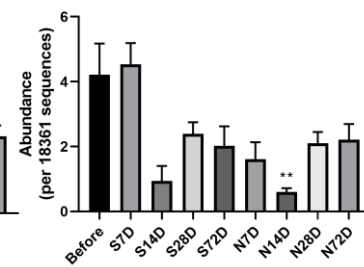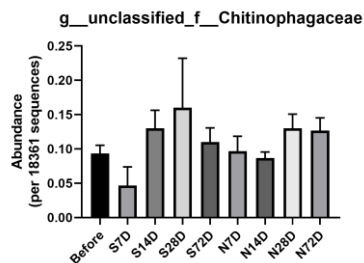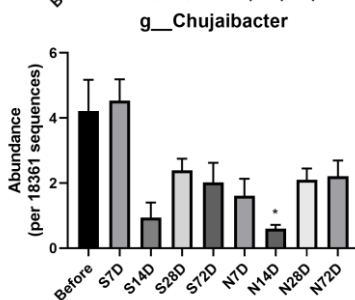

Supplement: Supplemental Information 5 [file peerj-09-12309-s005.pdf]

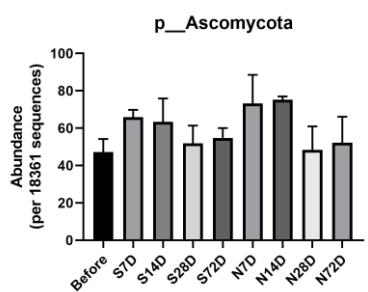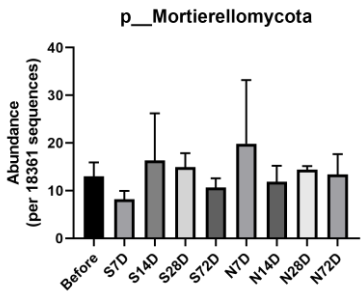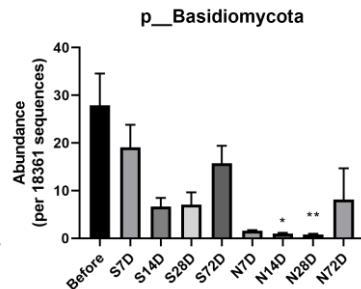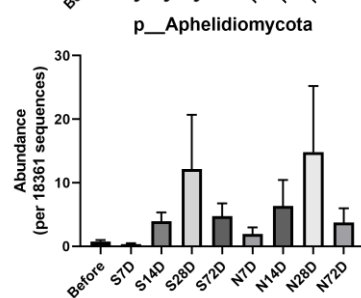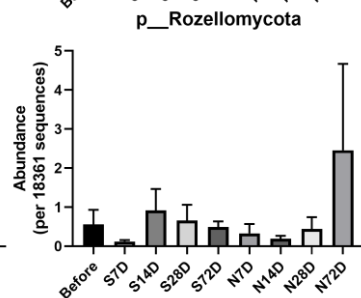

Supplement: Supplemental Information 6 [file peerj-09-12309-s006.pdf]

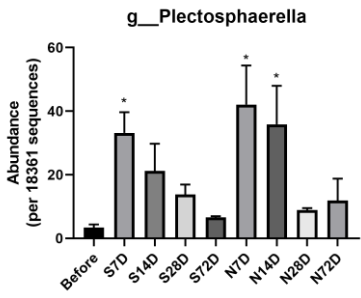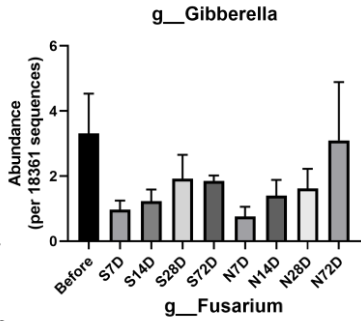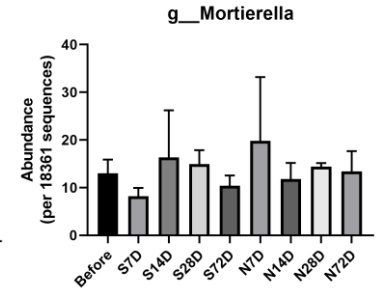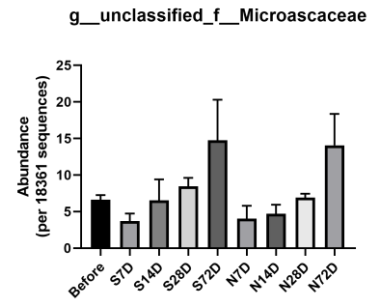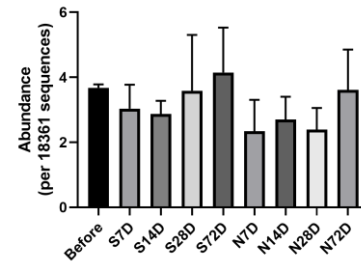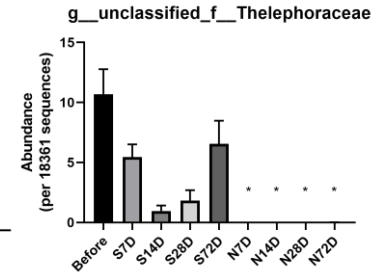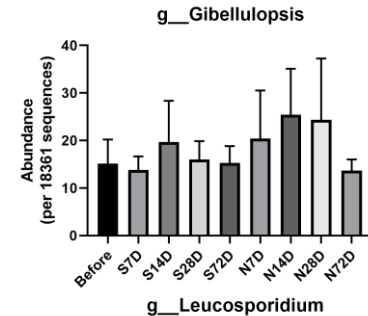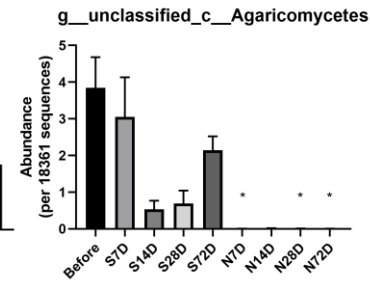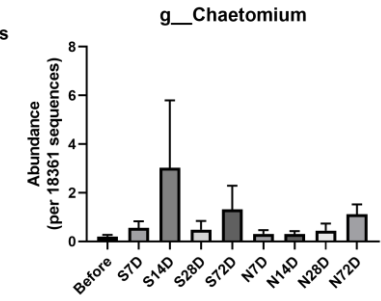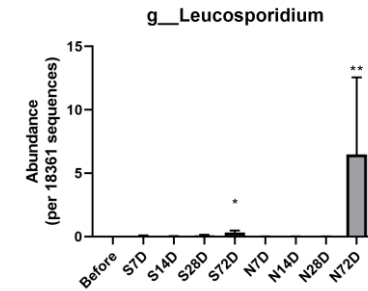

Supplement: Supplemental Information 7 [file peerj-09-12309-s007.pdf]

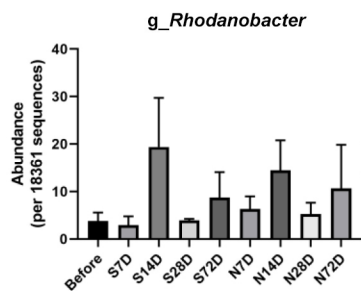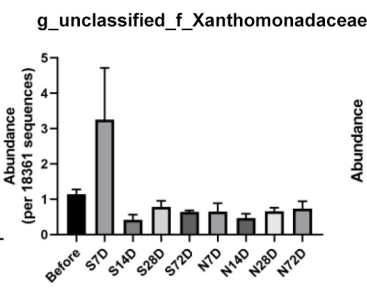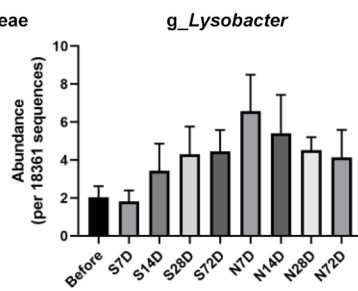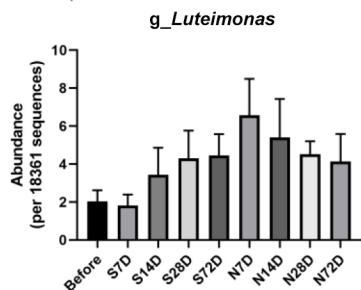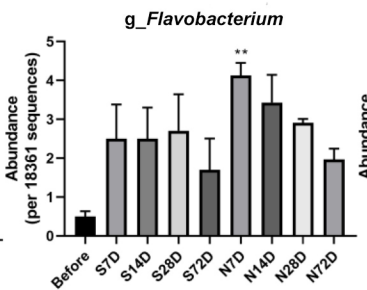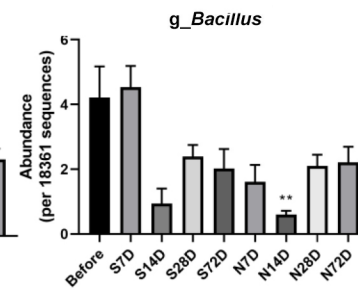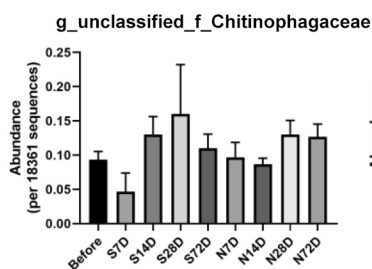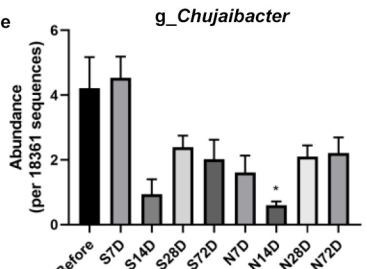

Supplement: Supplemental Information 8 [file peerj-09-12309-s008.pdf]

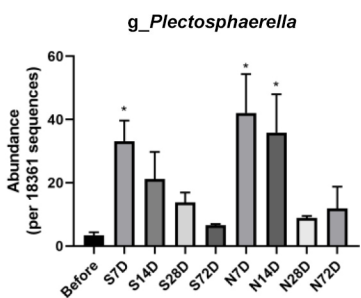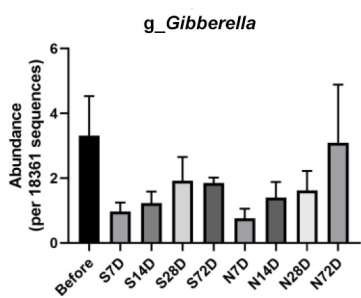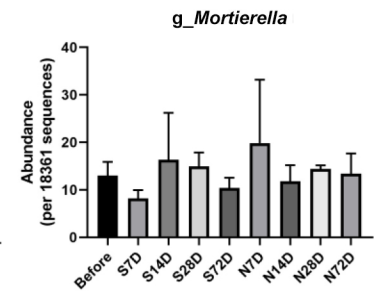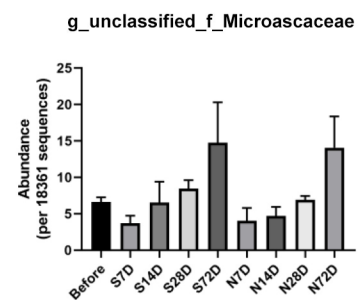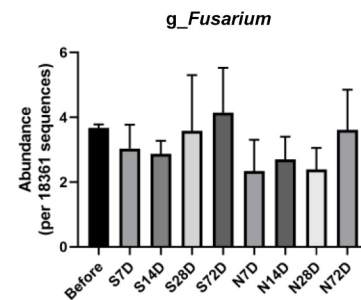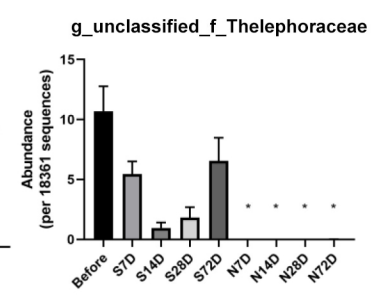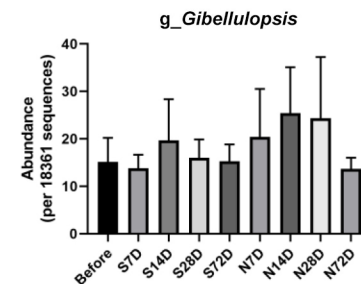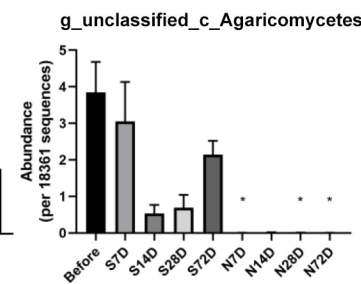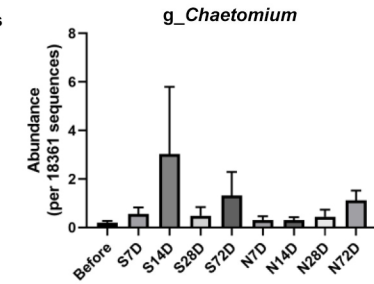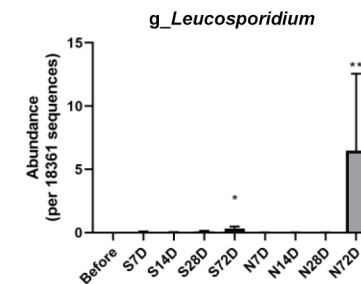

Supplement: Supplemental Information 9 [file peerj-09-12309-s009.pdf]
